# Premorbid Social Functioning and Affective Symptoms Predict Subjective Outcome Among Outpatients With Schizophrenia

**DOI:** 10.3389/fpsyt.2020.570857

**Published:** 2020-09-30

**Authors:** Christine M. Hoertnagl, Alexandra Kaufmann, Nursen Yalcin-Siedentopf, Nicole M. Pfaffenberger, Beatrice Frajo-Apor, Silvia Pardeller, Georg Kemmler, Alex Hofer

**Affiliations:** Department of Psychiatry, Psychotherapy and Psychosomatics, Division of Psychiatry I, Medical University Innsbruck, Innsbruck, Austria

**Keywords:** schizophrenia, psychopathology, premorbid functioning, side effects, life satisfaction, self-esteem, basic needs

## Abstract

Improving the subjective outcome of patients is an important target in the treatment of schizophrenia. Accordingly, the aim of the present study was to examine the association of factors deemed relevant in this context, i.e., premorbid functioning, residual symptoms, and side effects of antipsychotic medication, with subjective outcome. To this end, 70 clinically stable outpatients with schizophrenia were included into a cross-sectional study. Premorbid functioning, psychopathology, and side effects were assessed by using the Premorbid Adjustment Scale, the Positive and Negative Syndrome Scale, and the Udvalg for Kliniske Undersogelser Side Effect Rating Scale, respectively. Subjective outcome was measured in terms of life satisfaction (Life Satisfaction Questionnaire), self-esteem (Index of Self-Esteem), and needs for care (Berliner Bedürfnisinventar). Both premorbid social functioning and affective symptoms predicted life satisfaction, self-esteem, and patients’ basic needs, whereas positive and negative symptoms predicted needs in the health, social, and functional domains. Concerning side effects, parkinsonism and akathisia showed a significant negative correlation with self-esteem. These findings highlight the complex nature of subjective outcome in patients suffering from schizophrenia. Evidently, premorbid social functioning plays a prominent role in the experienced subjective outcome during the course of the illness. Furthermore, these preliminary findings underscore that constant efforts are essential to treat residual symptoms of the disorder and to avoid extrapyramidal motor side effects of antipsychotic medication. Longitudinal studies are needed to investigate this latter point in more detail.

## Introduction

Schizophrenia is a severe, disabling illness and is frequently associated with psychosocial disturbances including impairments in interpersonal relationships, independent living, and working ability (1–3) and consequently with a reduced subjective outcome. Nowadays, an optimal outcome in schizophrenia is redefined and includes clinical remission, an improved quality of life (QoL), life satisfaction as well as functional recovery. Generally, subjective perspectives such as patient-reported outcomes are increasingly utilized in a variety of medical fields, e.g., in oncology [e.g., (4)], in order to focus not only on clinical response to treatment but also, e.g., on side effects and their impact on subjective well-being and treatment adherence. Next to the side effects of treatment a failure to adequately treat symptoms has consistently been shown to be associated with poor subjective outcome such as QoL (5–11). A recent study by Phahladira and coworkers, for example, who followed 98 patients experiencing a first episode of schizophrenia spectrum disorders over 24 months of treatment showed that merely 9% of patients achieved both good QoL and functional remission despite not being in symptomatic remission (12). Findings on the associations between patients’ QoL and specific symptoms are divergent. While some studies reported on a negative impact of affective but not positive or negative symptoms (5, 13–15), others found that negative but not positive symptoms are linked to schizophrenia patients’ QoL (16). Altogether, a number of review articles have shown that the overall level of symptoms and specifically depressive symptoms are associated with lower QoL (13, 17). Moreover, Eack and Newhill noted that positive and negative symptoms were particularly related to poor QoL when investigating schizophrenia outpatients, whereas a negative relationship between general psychopathology and QoL was found across all study samples and treatment settings (13). With regard to negative symptoms Harvey et al. found a reduced emotional experience to be a stronger predictor of impairments in social outcome than emotional expression or the total negative symptom factor of the Positive and Negative Syndrome Scale [PANSS; (18, 19)].

Concerning the side effects of medication, we have previously shown that weight gain and parkinsonian symptoms as well as psychic side effects are of particular relevance in this context (5, 6).

Poor premorbid functioning has frequently been associated with a number of adverse aspects of schizophrenia including a poor outcome as well as a chronic clinical course and a higher severity of negative symptoms (20, 21). Walker and Lewine (22), for example, described that the premorbid functioning level impacts schizophrenia patients’ social outcome as well as residual symptomatology and response to treatment, and Browne and coworkers (23) detected a positive correlation between premorbid functioning and QoL in patients with first episode psychosis. However, studies investigating the association between premorbid functioning and differing subjective outcome domains are generally scarce since patients’ subjective outcome has frequently been equated with QoL, i.e., subjective satisfaction with specific areas of life and objective life circumstances (e.g., frequency of contacts with friends). Moreover, the definition of QoL varies and has often been equated with health-related domains (24). In an earlier study, we used the Lancashire Quality of Life Profile (25) and found a negative relationship between depression/anxiety, parkinsonism, and a negative attitude toward treatment and patients’ QoL, while cognitive symptoms and working capacity were associated with higher QoL (5). In order to expand on these previous findings and to focus on the long term management of individuals suffering from schizophrenia, the current study measured life satisfaction, self-esteem, and needs for care in clinically stable outpatients and investigated the associations between these issues and residual symptoms of the disorder, medication side effects, and premorbid functioning. Based on the findings of our earlier studies (5–7, 11) we hypothesized that depressed mood/anxiety, the presence of antipsychotic-induced side-effects as well as poor premorbid functioning would be negatively associated with all these subjective outcome domains.

## Materials and Methods

A convenience sample of 70 patients with schizophrenia or schizoaffective disorder (ICD-10: F20.x; ICD-10: F25.x) aged 18–60 from public outpatient mental health services were included into this cross-sectional study. At the time of investigation, clinical stability lasting for a period of at least 6 months had to be certified by the treating psychiatrist, i.e., all patients were treated as outpatients, there was no modification of treatment regimens, and psychopathology had not essentially changed during this period. The study had no impact on pharmacological treatment. Study participants signed informed consent forms in accordance with the ethics committee of the Medical University Innsbruck. They did not suffer from any other axis I disorder including substance abuse (assessed by self-report). Diagnoses were confirmed using chart information and reports from clinicians who had treated these patients. Ratings were completed by psychiatrists belonging to a trained schizophrenia research team. Next to the registration of sociodemographic data, various rating scales were applied, including the Global Assessment of Functioning Scale [GAF; (26)]. Additionally, all participants underwent cognitive assessments, which will be the subject of another report.

### Psychopathology

Psychopathology was rated by means of the PANSS (18). According to Lindenmayer et al. (27, 28), the PANSS was divided into five dimensions: (I) negative component (including the subscales “emotional withdrawal”, “poor rapport”, “passive social withdrawal”, “blunted affect”, “lack of spontaneity”, and “active social avoidance”), (II) excitement component (including the subscales “excitement”, “poor impulse control”, “uncooperativeness”, and “hostility”), (III) cognitive component (consisting of the subscales “conceptual disorganization”, “difficulty in abstract thinking”, “mannerisms and posturing”, “poor attention”, and “disorientation”), (IV) positive component (including the subscales “delusions”, “grandiosity”, “suspiciousness”, “hallucinatory behavior”, “stereotyped thinking”, and “unusual thought content”), and (V) depression/anxiety component (consisting of the subscales “depression”, “anxiety”, “guilt”, and “tension”).

### Side Effects

To quantify antipsychotic-induced side effects known to be related to subjective outcome parameters (parkinsonism, akathisia, sedation, weight gain, diminished sexual desire, and functional sexual disturbances), the Udvalg for Kliniske Undersogelser (UKU) Side Effect Rating Scale (29) was used. Each symptom is scored on a severity scale from 0–3, and the rater assesses whether the report can be attributed to a side effect (rated as improbable, possible, or probable) or is rather related to the disease. Only adverse effects with scores ≥1 on the above-mentioned UKU items and a possible or probable causal relationship with antipsychotic treatment were considered in the analysis.

### Premorbid Functioning

Premorbid functioning was quantified using the Premorbid Adjustment Scale [PAS; (30)], which is divided into five sections. The first four sections assess four time periods: childhood (up to age 11), early adolescence (age 12–15), late adolescence (age 16–18), and adulthood (age 19 and older). The fifth section provides an overall indication of premorbid educational, occupational, social, and energy level and was not used in the current study due to concerns regarding its validity (31).

The PAS covers two areas of functioning: academic functioning (achievements in school and adaption to school) and social functioning [sociability/withdrawal, peer relationships, and ability to establish interpersonal and sexual relationships (starting at age 12)]. Items are scored on a scale from 0 to 6, with 0 denoting the highest level of functioning and 6 the lowest. The range of scoring for each developmental period is the same, allowing for comparison scores across developmental periods. For the purpose of subsequent statistical analysis, summary scores for academic functioning and social functioning were calculated.

Andreasen et al. (32) defined the premorbid period to end one year before the first admission to hospital or the onset of florid psychotic symptoms. Accordingly, the PAS was completed only for the developmental stages preceding the onset of illness.

### Subjective Outcome

#### Life Satisfaction

The Life Satisfaction Questionnaire [LSQ; (33)] comprises the following life domains: health, work, finances, leisure, partnership, relation to own children, own person, sexuality, friends/relatives, and residence. Items are scored on a seven-point scale ranging from completely dissatisfied (1) to completely satisfied (7), with 4 (alternatively satisfied and dissatisfied) as a midpoint. Subscores for satisfaction with individual life domains result from averaging the individual satisfaction scores within domains. Due to the fact that most subjects included into the study were single and childless, these life domains (partnership, children) could not be considered in the analysis. In addition to the subscores a total score was calculated as the sum of all subscores (except partnership and children).

#### Self-Esteem

Self-esteem was assessed using the Index of Self-Esteem [ISE; (34)], a 25-item self-report measure with a 7-point Likert-type scale which targets the subjective evaluation of self. Responses vary from “none of the time” to “all of the time”. The raw scores of the ISE were converted to a range from 0 to 100 by linear transformation (0 = best possible score, 100 = worst possible score) with scores below 30 representing high self-esteem, while scores above 30 represent low self-esteem.

#### Needs for Care

We used the Berliner Bedürfnisinventar [BeBI; (35)], Patient Version, which is similar to the Camberwell Assessment of Need [CAN; (36)], to assess patients’ needs. The BeBI comprises 15 individual areas which are grouped into four domains of needs: health (including physical health, mental health, information, drugs/alcohol, safety), basic (including accommodation and food), social (including company, intimate relationships, and sexual expression), and functioning (including looking after home, work/occupation, self-care, finances, and money). Each item of the BeBI comprises the same structure. The first question asks whether a need exists. If there is no need in any particular area, the interviewer proceeds straight to the next item. If a met or unmet need does exist, further questions investigating how much care is received from relatives/friends or formal services are asked (0 = no help, 1 = low help, 2 = moderate help, 3 = high help). For the purposes of this study only information about whether a need existed was used. Summary scores for the four needs domains (health, basic, social, and functioning) were calculated by taking the means of the scores for the respective areas (0 = no needs, 1 = highest possible needs score). A total needs score was formed by averaging over all areas.

### Statistical Methods

Main purpose of the statistical analysis was an investigation of the effects of premorbid functioning (PAS) and psychopathology (PANSS) on subjective outcome (life satisfaction, self-esteem, and needs for care). For completeness, the effects of antipsychotic-induced side effects (UKU) on outcome were also taken into account. In the first part of the analysis correlation analyses were performed. Spearman rank correlation coefficients were used for this purpose as the majority of the variables involved showed significant departures from normality (tested by means of the Shapiro-Wilk test). To avoid problems with extensive multiple testing, a multivariate pre-test by means of canonical correlation was performed between blocks of variables (e.g., PAS subscales with LSQ subscales). Only if this analysis yielded a statistically significant first canonical variate the corresponding correlation matrix was analyzed.

The second part of the analysis consisted of multiple linear regression analyses with premorbid functioning (PAS subscales) and psychopathology (PANSS components) as independent variables and subjective outcome as dependent variable. To limit the number of regression analyses to be performed, the subscales of the LSQ were summarized to two factors: personal/social (comprising health, own person, sexuality, and friends/relatives) and functional (work, leisure, finances, and residence) life satisfaction. Each regression analysis was divided into two steps. In the first instance, only the effect of psychopathology (as a measure of present disease severity) on treatment outcome was considered. In a second step, the additional effect of premorbid functioning was taken into account. Significant predictors were determined by forward variable selection. In order to adhere to a subjects to variables ratio of approximately 10 the number of independent variables was limited to 7 (see *Power Analysis*). The measure R² was used to describe the proportion of variance accounted for by the regression model. All statistical tests were performed at a 0.05 level of significance.

### Power Analysis

The sample size of 70 schizophrenia patients is sufficient to detect, under standard conditions regarding type-one error (two-tailed α = 0.05) and power (1-β = 0.8), Pearson correlations or Spearman rank correlations of r = 0.328 or greater. This is a medium effect size according to Cohen’s classification (37). Moreover, under the same conditions as above, the sample size of 70 allows detection of an effect size of f² = 0.116 in a linear regression analysis with up to 7 independent variables, when testing for the effect of one additional predictor, which is again a medium effect size. The number of 7 independent variables should be regarded as an upper limit. According to recommendations for multiple linear regression, the subjects per variable ratio should be at least 9.4:1 if the expected R² is in the medium range (R² = 0.35), or 11.6:1 if the expected R² is slightly lower (R² = 0.3), resulting in a maximum of 7 or 6 independent variables, respectively (38). More details may be found in the cited reference.

## Results

### Patient Characteristics

**Table 1** summarizes demographic and clinical characteristics of the study sample. The majority of patients were male, their mean age was 43 years with a mean duration of illness of approximately 14 years. In terms of the Lindenmayer five-factor model, negative symptoms showed the highest mean score followed by depression/anxiety. Scores for both positive and cognitive symptoms as well as excitement were generally low. Most patients received monotherapy with a second generation antipsychotic.

**Table 1:** Patient characteristics.

Patients, N	70
Age, mean ± SD, y	43.3 ± 8.9
Sex, %, M/F	58.6/41.4
Duration of illness, mean ± SD, y	14.4 ± 8.3
Education, mean ± SD, y	11.7 ± 3.8
	
GAF score, mean ± SD	61.3 ± 17.7
	
PANSS score, mean ± SD	
Total score (range: 30–120)	54.7 ± 18.8
	
Subscores^a^ (range: 1–7)	Negative: 2.31 ± 1.24
	Excitement: 1.20 ± 0.43
	Cognitive: 1.69 ± 0.81
	Positive: 1.76 ± 0.81
	Depression/anxiety: 1.87 ± 0.83
	
Antipsychotic treatment, N (%)	4 (5.7) FGA (monotherapy)
	59 (84.3) SGA (monotherapy)
	2 (2.9) FGA + SGA (combined treatment)
	5 (7.1) SGA + SGA (combined treatment)
	
Housing, N (%)	With original family: 12 (17.1)
	With own family: 11 (15.7)
	Alone: 36 (51.4)
	In a small group home: 11 (15.7)
	
Partnership status, N (%)	Single: 53 (75.7)
	Married/stable partnership: 6 (8.6)
	Divorced/widowed: 11 (15.7)
	
Employment status, N (%)	Full time employment: 3 (4.3)
	Part time employment: 3 (4.3)
	Supported employment: 10 (14.3)
Diagnosis	Schizophrenia: 65 (92.9) Schizoaffective disorder: 5 (7.1)

GAF, Global Assessment of Functioning Scale; PANSS, Positive and Negative Syndrome Scale; FGA, first-generation antipsychotic; SGA, second-generation antipsychotic.

a Subscores as defined by the Lindenmayer five-factor model.

### Antipsychotic-Induced Side Effects

According to the UKU items used, the most frequently reported antipsychotic-induced side-effects included weight gain (52.9%) and parkinsonism (50.0%). Moreover, diminished sexual desire was observed in 42.6% of patients and sedation in 41.4%. Functional sexual disturbances were assessed in 37.2% of patients, akathisia in 11.8%.

### Premorbid Functioning

Premorbid academic functioning deteriorated significantly from childhood (1.69 ± 1.11) to early adolescence (2.60 ± 1.17; Wilcoxon test, Z = 2.09, p = 0.036) and remained constant thereafter (late adolescence: 2.12 ± 1.31). The PAS academic functioning summary score amounted to 1.71 ± 0.88.

Premorbid social functioning deteriorated significantly from childhood (1.32 ± 1.41) to early adolescence (1.55 ± 1.32; Z = 2.14, p = 0.032), remained constant from early to late adolescence (1.63 ± 1.34), and deteriorated again from late adolescence to adulthood (2.06 ± 1.23; Z = 3.84, p < 0.001). The PAS social functioning summary score amounted to 1.71 ± 1.03.

### Life Satisfaction

With a mean LSQ total score of 5.19 ± 0.67 patients’ global life satisfaction was generally positive. Of the life domains assessed, “residence” received the highest satisfaction ratings (5.87 ± 0.67) followed by “leisure” (5.59 ± 0.81), while “health” (4.87 ± 0.94) and “sexuality” (4.60 ± 1.12) had the lowest ratings.

### Self-Esteem

The mean ISE score on the 0–100 scale amounted to 34.69 ± 16.03. 64.3% of patients had a score ≥30 and thus demonstrated low self-esteem.

### Needs for Care

Of the four needs domains assessed, patients indicated fewest social needs (0.17 ± 0.25) followed by needs in the areas of health (0.25 ± 0.17) and functioning (0.31 ± 0.28). Most needs were indicated in the basic domain (0.36 ± 0.41). The mean total needs score amounted to 0.27 ± 0.18. With regard to individual areas, most patients indicated to have existing needs in the areas of mental health (74.3%), accommodation (48.6%), work/occupation (46.4%), looking after home, and finances (41.4%, each).

### Results of Canonical Correlation Analyses

Canonical correlation analyses revealed a significant relationship between the PANSS components and all subjective outcome measures, i.e., LSQ, ISE, and BeBI (details in **Tables 2**, **3**) and likewise between the PAS and all subjective outcome scales (details in **Tables 4**, **5**). However, for the UKU only the relationship with the ISE attained statistical significance (r_canonical_ = 0.439; Wilk’s λ = 0.807; F = 2.87; d.f. = 5.60; p = 0.022), whereas the relationship between the UKU and both the LSQ and the BeBI failed to reach statistical significance (p > 0.1 in both cases). We therefore performed correlation analyses for the PANSS and PAS with all subjective outcome measures, whereas for the UKU only correlation analyses with the ISE were performed.

**Table 2:** Association of psychopathology with life satisfaction and self-esteem (Spearman rank correlation).

Subjective Outcome	PANSS dimensions (Lindenmayer)					
	PANSS Positive	PANSS Negative	PANSS Depression/Anxiety	PANSS Cognitive	PANSS Excitement	PANSS Total score
*Life satisfaction (LSQ)^a^*						
Healthiness	−.276*	**−**.139	−.526***	.004	.011	-.252*
Work	**−**.220	**−**.119	−.297*	**−**.087	**−**.066	**−**.200
Finances	**−**.222	**−**.062	**−**.220	.132	.026	**−**.096
Leisure	**−**.201	**−**.172	−.345**	**−**.073	.038	**−**.203
Own person	−.306**	**−**.134	−.432***	**−**.064	.062	**−**.221
Sexuality	−.306*	**−**.186	−.295**	**−**.093	.051	−.241*
Friends/relatives	−.355**	**−**.229	−.399***	.004	.010	−.271*
Residence	−.243*	**−**.214	−.272*	**−**.087	.113	**−**.224
FLZ Total	−.416***	−.291*	−.504***	**−**.074	.096	−.358**
*Self-esteem (ISE)^b^*						
ISE Total score	.213	.332**	.425***	.047	**−**.071	.290*

PANSS, Positive and Negative Syndrome Scale; LSQ, Life Satisfaction Questionnaire; ISE, Index of self-esteem.

a Canonical correlation of FLZ with PANSS: r(canonical) = 0.699; Wilk’s λ = 0.351; F = 1.50; df = 40, 220; p = 0.037.

b Canonical correlation of ISE with PANSS: r(canonical) = 0.581; Wilk’s λ = 0.663; F = 6.52; df = 5, 64; p < 0.001.

*p < 0.05, **p < 0.01, ***p < 0.001.Bold print indicates statistically significant correlations.

**Table 3:** Association of psychopathology with needs for care (Spearman rank correlation).

Needs for care (BeBI)^a^	PANSS dimensions (Lindenmayer)					
	PANSS Positive	PANSS Negative	PANSS Depression/Anxiety	PANSS Cognitive	PANSS Excitement	PANSSTotal score
Basic needs	.298*	.172	.263*	.050	.129	.254*
Health needs	.270*	.119	.426**	.136	.190	.278*
Social needs	.441***	.469***	.342**	.388***	.234	.533***
Functional needs	.416***	.439***	.266*	.251*	.056	.469***
Total Needs	.507***	.412***	.431***	.275*	.166	.531***

PANSS, Positive and Negative Syndrome Scale; BeBi, Berliner Bedürfnisinventar.

a Canonical correlation of BeBI with PANSS: r(canonical) = 0.611; Wilk’s λ = 0.482, F = 2.50, df = 20, 203; p = 0.001.

*p < 0.05, **p < 0.01, ***p < 0.001.Bold print indicates statistically significant correlations.

**Table 4:** Association of premorbid functioning with life satisfaction and self-esteem (Spearman rank correlation).

Subjective Outcome	Premorbid functioning(PAS summary scores)^a^		
	Academic		Social
*Life satisfaction (LSQ)^b^*			
Healthiness	.002		−.301*
Work	−.077		−.122
Finances	−.085		−.045
Leisure	−.293*		−.095
Own person	−.109		−.391***
Sexuality	−.258*		−.317**
Friends/relatives	−.217		−.261*
Residence	−.215		−.297*
Total	−.269*		−.429**
*Self-esteem (ISE)^c^^,^^d^*			
ISE Total	.249*		.520***

PAS, Premorbid Adjustment Scale; LSQ, Life Satisfaction Questionnaire; ISE, Index of self-esteem.

a High scores in the PAS indicate poor premorbid adjustment.

b Canonical correlation of FLZ with PAS: r(canonical) = 0.543; Wilk’s λ = 0.624; F = 1.76; df = 16, 106; p = 0.047.

c High scores in the ISE indicate low self-esteem.

d Canonical correlation of ISE with PAS: r(canonical) = 0.541; Wilk’s λ = 0.707; F = 13.9; df = 2, 67; p < 0.001.

*p < 0.05, **p < 0.01, ***p < 0.001. Bold print indicates statistically significant correlations.

**Table 5:** Association of premorbid functioning with needs for care (Spearman rank correlation).

Needs for care (BeBI) Domain^b^	Premorbid functioning(PAS summary scores)^a^	
	Academic	Social
Basic needs	−.079	.175
Health needs	.187	.290*
Social needs	.077	327**
Functional needs	.088	.271*
Total Needs	.105	.353**

PAS, Premorbid Adjustment Scale; BeBi, Berliner Bedürfnisinventar.

a High scores in the PAS indicate poor premorbid adjustment.

b Canonical correlation of BeBI with PAS: r(canonical) = 0.434; Wilk’s λ = 0.776; F = 2.16; df = 8, 203; p = 0.035.

*p < 0.05, **p < 0.01. Bold print indicates statistically significant correlations.

### Results of Spearman Rank Correlation

Among psychopathological symptoms, depression/anxiety was negatively associated with all life domains assessed by the LSQ except “finances” as well as with the LSQ total score. A further negative association was shown with self-esteem, a positive association with all domains of the BeBI. The positive component of the PANSS showed a negative correlation with most domains assessed by the LSQ as well as with the LSQ total score and a positive correlation with all domains of the BeBI. No correlation was found between positive symptoms and self-esteem. On the other hand, negative symptoms were negatively associated with the LSQ total score (but not with individual life domains) and with self-esteem. Both the negative and the cognitive components of the PANSS showed a positive correlation with social and functional needs as well as with the BeBI total score. Cognitive symptoms were not associated with life satisfaction or self-esteem. In addition, we found no correlation between the excitement component of the PANSS and all subjective outcome scales. The PANSS total score showed a negative correlation with the health, sexuality, and friends/relatives domains of the LSQ, the LSQ total score, and self-esteem. Furthermore, we found a positive correlation with all domains of the BeBI.

Two of the UKU items showed a significant correlation with self-esteem, assessed with the ISE (higher scores denote lower self-esteem): parkinsonism (r_Spearman_ = 0.244, p = 0.042) and akathisia (r_Spearman_ = 0.325, p = 0.007). None of the other UKU items showed a significant correlation with the ISE.

Premorbid school functioning was negatively correlated with the leisure and sexuality domains of the LSQ, the LSQ total score and self-esteem, but we found no correlation with patients’ needs. On the other hand, premorbid social functioning was negatively correlated with most life domains assessed by the LSQ, the LSQ total score, and self-esteem and positively correlated with health, social, and functional needs and the BeBI total score.

### Results of Multiple Linear Regression Analysis

The combined effect of psychopathology and premorbid functioning on subjective outcome was analyzed by multiple linear regression analysis (see **Table 6**). To limit the number of independent variables, patient characteristics (age, sex, duration if illness) were not included in the regression analysis. However, none of them showed a significant correlation with any of the dependent variables. Regarding patients’ *life satisfaction* both the PANSS (subscale depression/anxiety) and the PAS (social functioning score) independently contributed to the prediction of the LSQ total score. The same pattern was observed for the personal/social factor of the LSQ, whereas only the PANSS, but not the PAS, contributed significantly to the prediction of the functional factor. Both the PANSS depression/anxiety subscale and the PAS social functioning score were found to be significant predictors of patients’ *self-esteem*.

**Table 6:** Prediction of subjective outcome by psychopathology and premorbid adjustment (multiple linear regression analysis).

Outcome measure	Prediction by PANSS dimensions (Lindenmayer) alone				Improvement of prediction by adding premorbid functioning (PAS)				Total R²
	Predictor	Beta	p	R²	Predictor	Beta	p	δR²	
*Life satisfaction (LSQ)*									
Factor I: personal/social^a^	Depression/anxiety	−.524	<.001	.274	Social functioning^c^	.243	.030	.050	.324
Factor II: functional^b^	Depression/anxiety	−.320	.008	.102	–	–	–	–	.102
Total score	Depression/anxiety	−.518	<.001	.243	Social functioning^c^	.267	.022	.059	.302
*Self-esteem (ISE)*									
ISE total score	Depression/anxiety	−.506	<.001	.256	Social functioning^c^	.405	<.001	.139	.395
*Needs for care (BeBI)*									
Basic needs	Depression/anxiety	.239	.046	.057	Social functioning^c^	.323	.006	.062	.119
Health needs	Depression/anxiety	.364	.002	.132	–	–	–	–	.132
Social needs	Positive symptoms Negative symptoms	.299 .279	<.001	.230	–	–	–	–	.230
Functional needs	Negative symptoms	.496	<.001	.246	–	–	–	–	.246
Total needs	Positive symptoms Negative symptoms	.296 .295	<.001	.239	–	–	–	–	.239

PANSS, Positive and Negative Syndrome Scale; PAS, Premorbid Adjustment Scale; LSQ, Life Satisfaction Questionnaire; ISE, Index of self-esteem; BeBI, Berliner. Bedürfnisinventar

a comprises the domains healthiness, own person, sexuality, and friends/relatives.

b comprises the domains work, leisure, finances, and residence.

c comprises premorbid social adjustment in childhood, early adolescence, late adolescence, and adulthood.

With regard to *needs for care* both the PANSS positive symptoms and negative symptoms components showed a significant effect on total needs, whereas no significant effect of premorbid functioning was seen after adjustment for the PANSS. Similar findings were obtained for social needs and functional needs. Only one PANSS component (depression/anxiety) significantly contributed to the prediction of health needs, whereas basic needs (comprising the areas of accommodation and food) were predicted by a combination of the PANSS (depression/anxiety component) and the PAS (social functioning score).

## Discussion

Our sample consisted of chronically ill outpatients with merely mild symptoms (39). They had been stable both from a symptomatic and a medication perspective before study inclusion and accordingly, we were able to study the persistent impairments associated with schizophrenia spectrum disorders. We could show that affective symptoms and premorbid social functioning adversely influenced patients’ life satisfaction, self-esteem, and basic needs in a multiple linear regression analysis. In addition, affective symptoms predicted needs concerning health, negative symptoms predicted functional needs and both positive and negative symptoms predicted social needs.

Given the basic medical service and the well-developed network of social benefits in Austria it is not surprising that corresponding to our previous findings in independent samples (5, 6) study participants were satisfied with their lives in general and that functional life issues like “residence” or “leisure”, which have recently been shown to destigmatize patients with schizophrenia (40), reached the highest ratings in the LSQ. A number of previous studies have shown that older age is associated with higher life satisfaction [e.g., (5, 41)]. The sample investigated in this study was middle-aged and had an average duration of illness of 14 years. We therefore hypothesize that study participants may have developed coping strategies to deal with the disorder and to adjust to their health condition and to available resources, which, in turn, may have had a positive impact on life satisfaction. On the other hand and similar to previous studies (6, 42–46), the majority of our sample demonstrated low self-esteem. In addition, more than 40% of patients indicated to have existing needs in the domains of accommodation, work/occupation, looking after home, and finances, and approximately three quarters expressed a need concerning mental health. Notably, support in mental health has been shown to lead to insight into the illness and has been associated with adherence to the treatment (47). Accordingly, our findings are in line with the suggestion that even during periods of clinical stability continuous psychosocial support is needed (48).

The most frequently reported side-effects of antipsychotic treatment were similar to those found in other studies (49–51) and included weight gain, parkinsonism, diminished sexual desire, functional sexual disturbances, and sedation. They definitely play a prominent role in treatment adherence (52), however, with the exception of a negative association between extrapyramidal symptoms and patients’ self-esteem the relationship between side effects and subjective outcome parameters failed to reach statistical significance, which may be a reflection of sample selection. In the light of previous findings this result may partly also be attributable to the fact that most study participants were treated with second generation antipsychotics. Voruganti and coworkers, for example, detected a significant improvement in patients’ QoL after they were switched from first to second generation antipsychotics (53), and Karamatskos and coworkers suggested that such a switch may reduce negative symptoms and eventually contribute to a higher subjective well-being as well as a better medication adherence and thereby to an improved outcome (51). However, this issue cannot be addressed by our data.

Residual positive and affective symptoms were associated with less life satisfaction and a higher degree of patients’ needs, whereas both negative and affective symptoms were negatively associated with self-esteem. The latter finding corroborates that of some previous studies (46, 54), however, still other investigations found that self-esteem is especially related to positive symptoms (55). Again, these divergent results may be a reflection of sample selection and still other issues, e.g., cultural aspects, may also be relevant in this context. Our finding of residual affective symptoms being negatively associated with most life domains assessed by the LSQ and general life satisfaction is in agreement with previous reports (5, 9, 56–58). However, the PANSS is not ideal to assess affective symptoms in schizophrenia patients and future studies should therefore use specific instruments to investigate the association between depression/anxiety and life satisfaction in more detail.

Several previous studies indicated that next to affective symptoms patients’ QoL is primarily related to negative symptoms (59, 60), while others have shown that positive symptoms may be relevant in this context as well (6, 58, 61, 62). Norman and coworkers suggested that these divergent findings may be attributable to the questionnaires used. They noted that reports suggesting that QoL is primarily related to negative rather than positive symptoms frequently used the Quality of Life Scale which focuses on deficit symptoms and does not assess subjective general wellbeing, whereas in studies using the General Well-Being Scale QoL was primarily related to the severity of positive symptoms (63). The LSQ used in the current study measures a wide spectrum of domains, including several aspects of well-being. Accordingly, our finding of an association between the LSQ total score and positive, negative as well as affective symptoms is not surprising.

Study participants presented with deteriorating premorbid functioning, which is a common characteristic of schizophrenia (64–66). Premorbid social functioning correlated with all subjective outcome parameters assessed in this study, whereas premorbid academic functioning correlated with life satisfaction and self-esteem, but not with patients’ needs. Intuitively, these results make sense, however, causal relationships cannot be deduced from the findings of this study due to its cross-sectional design.

In summary, our findings suggest that the strengthening of social adjustment during the prodromal phase as well as the improvement of affective symptoms may promote the subjective outcome of patients suffering from schizophrenia. Notwithstanding the implications of these findings, there are a number of limitations that should be considered. First, we applied a cross-sectional design and included a relatively small sample of mildly ill outpatients (reflected by low PANSS and high GAF scores) who were adherent to pharmacological and psychological treatment. This limits the generalizability of the obtained results. As a matter of course, a cross-sectional study cannot determine whether an “outcome” results from treatment, from the natural outcome of the illness, or perhaps from personality or other factors, e.g., patients’ resilience, internalized stigma, or religiosity (6, 45, 67). Longitudinal designs clearly allow a more rigorous investigation of potentially causal relationships between the assessed variables and their influence on subjective outcome. Such studies could, for example, clarify whether residual symptoms or side effects are actually influencing life satisfaction after patients are switched from one antipsychotic drug to another. Accordingly, it will be critical to collect follow-up data to investigate how influencing variables and consequently patients’ subjective outcome change over time. Secondly, we did not collect any collateral information from family members regarding patients’ premorbid functioning. Despite with schizophrenia having been shown to be as reliable as healthy subjects when reporting on premorbid functioning (68) it is conceivable that they may not have remembered correctly. Thirdly, subjective outcome was obviously self-reported, which can result in social desirability bias. Lastly, some of the scales used in this study were not designed to specifically evaluate patients with schizophrenia. Notwithstanding these limitations, our findings reemphasize the relevance of promoting the subjective outcome of patients as part of a comprehensive treatment of schizophrenia. As mentioned above, further longitudinal studies including patients when starting treatment with an antipsychotic drug and following them up are needed to investigate the impact of changes in psychopathology as well as single side effects on subjective outcome parameters in the long-term.

## Data Availability Statement

All datasets presented in this study are included in the article/supplementary material.

## Ethics Statement

The studies involving human participants were reviewed and approved by Ethics committee of the University of Innsbruck, Austria. The patients/participants provided their written informed consent to participate in this study.

## Author Contributions

Concept and design: all authors. Acquisition of data: CH, AK, NY-S, NP, and BF-A. Analysis and interpretation of data: all authors. Drafting the article: CH. Revision: AK, NY-S, NP, BF-A, GK, and AH. All authors contributed to the article and approved the submitted version.

## Conflict of Interest

The authors declare that the research was conducted in the absence of any commercial or financial relationships that could be construed as a potential conflict of interest.
